# Parents’ and Teachers’ Perceptions of Risks Associated with Children’s Walks to School in Blantyre, Malawi

**DOI:** 10.3390/ijerph21111479

**Published:** 2024-11-07

**Authors:** Bosco Chinkonda, Alejandra Piragauta, Dennis Mazingi, Linda Chokotho, Monica Nzanga, Steve Manyozo, Prasanthi Puvanachandra, Margaret Peden

**Affiliations:** 1 Department of Public Health, Kamuzu University of Health Sciences (KUHeS), Blantyre P.O. Box 360, Malawi; boscochinkonda@gmail.com (B.C.); lindachokotho@gmail.com (L.C.);; 2 The George Institute for Global Health UK, London W12 7RZ, UKmpeden@georgeinstitute.org.uk (M.P.); 3 Academy of Medical Sciences, Malawi University of Science and Technology (MUST), Limbe P.O. Box 5196, Malawi; 4 Imperial College London, School of Public Health, London W12 0BZ, UK

**Keywords:** children, road safety, air pollution, schools, focus groups

## Abstract

(1) Background: This study explored the challenges faced by schoolchildren while commuting to school, particularly the hazards associated with poor road conditions, exposure to high-speed traffic, and traffic-related air pollution (TRAP). (2) Methods: The research focused on community perspectives gathered through four focus group discussions (FGDs) involving parents and teachers from two primary schools in Blantyre City. Employing qualitative analysis with NVivo, themes, sub-themes, and codes were developed collaboratively within the research team. (3) Results: The community identified road safety risks, emphasizing concerns about different actors’ risky behaviors, poor infrastructure (such as sidewalks, crossings, and signals), and personal safety issues. Proposed solutions for road safety involved educational initiatives for students, infrastructure enhancement, and enforcing stricter penalties. The study revealed a lack of awareness about air pollution among participants, which they often associated solely with unpleasant odors. Suggestions for addressing air pollution centered on educational interventions related to waste disposal and hygiene practices. (4) Conclusions: This research underscores the community’s adeptness at identifying road safety issues and proposing viable interventions. However, it highlights the need for enhanced education and awareness regarding air pollution. The paper advocates for community engagement to comprehensively address these challenges, fostering community cohesion, and empowering community members to advocate for change.

## 1. Introduction

Road traffic injuries (RTIs) and deaths continue to pose a global health challenge and development issue. According to the World Health Organization (WHO) Global Status Report on Road Safety 2023, approximately 1.19 million people died in road traffic collisions during 2021, corresponding to a rate of 15 road traffic deaths per 100,000 people worldwide [1]. Low- and middle-income countries (LMICs) bore the brunt, accounting for 92% of these road traffic fatalities [1], and the African region has the highest fatality rate at 19 road traffic deaths per 100,000 people [1]. The risk of death is three times higher in low-income countries (LICs) than in high-income countries despite these countries having less than 1% of all motor vehicles [1]. Mortality rates are high in LICs because of multiple factors including poor road infrastructure, the limited enforcement of traffic laws, poor vehicle standards, outdated urban planning, and poor road user behavior [1,2].

RTIs constitute the leading cause of death for children and youth worldwide. These incidents not only account for most fatalities among children and adolescents aged 5 to 29 years but also contribute significantly to long-term disability, imposing substantial costs on societies [1,2]. Furthermore, resource-limited settings are disproportionately affected, with 93% of child road traffic deaths occurring in LMICs [2].

In addition to deaths, life-changing injuries, and long-lasting health problems caused by road traffic collisions [2], children in urban areas are also affected by insidious damage to their developing lungs and brains by breathing traffic-related air pollutants (TRAPs). Exposure to air pollutants has been linked to growth stunting, cognitive development delays, and chronic respiratory problems [3,4]. However, the extent and specific health consequences of air pollutant exposure remain unclear. While air pollution’s adverse effects on children are evident, these impacts are not universally consistent [5], with limited evidence available from contexts similar to Malawi.

An estimated 350,000 children and adolescents are killed every year in road traffic crashes or by the effects of urban outdoor air pollution, to which road traffic is a significant contributing factor [1]. Air pollution exposure is linked to road safety factors such as the proximity of the footpath to the road and the time spent walking close to busy roads [6,7,8]. There are, therefore, significant opportunities for complementary mitigation measures to drive improvements in child health, safety, and well-being.

The road traffic death rate in Malawi in 2019 was 33.4 deaths per 100,000 people, among the highest in the world [9], and the level of fine particulate matter (PM_2.5_) was at least double the WHO threshold of 10 µg/m^3^ [10]. Pedestrians account for half of the road traffic deaths in the country and research shows that there has been a drastic increase in the number of RTIs in the last decade, particularly among children and adolescents, who represent 53.8% of those admitted to hospitals [11]. Many children in Malawi walk to school on roads in poor condition that are dangerous and heavily polluted. Some schools are even located along major highways, which increase both exposure to high-speed traffic and high levels of TRAPs [12]. Additionally, compared to adults, children are more susceptible to road fatalities due to factors such as their limited impulse control, slower reaction times, and poorer perceptions of risk [13].

Several affordable interventions could be put in place to reduce the adverse effects on road safety and air pollution [14]. Following a Safe System Approach, actions could include one or a combination of the following: policy changes focused on reducing speed limits around schools and controlling vehicle emissions, infrastructure modifications, and educational or behavior change components [15]. Some of these approaches have already been applied in neighboring Tanzania, where the nongovernmental organization (NGO) AMEND has implemented a School Area Road Safety Assessment and Improvement (SARSAI) project demonstrating the feasibility and effectiveness of implementing a set of complex interventions to reduce child pedestrian collisions [16]. However, less is known about reducing children’s exposure to poor air quality in less-resourced settings although significant work has been carried out in this regard in London [17]. To our knowledge, no studies have been conducted that have simultaneously addressed road safety and air pollution among school-going children anywhere in Africa.

This study was therefore conducted to document the community’s perspective regarding road safety and air pollution risks that children face on their journeys to and from school. This research was part of the Risk Elimination on Walks to School (RemWalkS) study funded by the UK Medical Research Council (grant number MR/W004348/1), which aimed to develop a set of interventions designed to reduce both the risk of injury and exposure to poor air quality on walks to school among 12–16-year-olds in Blantyre, Malawi. This research sought to answer the following question: What are communities’ perceptions of the risks and facilitators of road traffic injuries and air pollution for children on their journey to school?

## 2. Materials and Methods

### 2.1. Study Design

As part of the mixed-methods RemWalkS study, this project component employed a qualitative study design, which allowed for a deeper understanding of the phenomena [18]. Taking the form of four focus group discussions (FGDs), the study provided space to generate a contextualized understanding and triangulation of information on road safety and air pollution risk factors children face on their journeys to school.

### 2.2. Study Setting

Participants were selected from two different primary schools in Blantyre, Malawi, namely Chirimba Primary School in the peri-urban area and Kanjedza Primary School in the urban area. Both schools are located within Blantyre City, one of the two large cities in Malawi. Despite their being in the same city, the selection of the two schools was meant to ensure peri-urban and urban representation of the city corresponding to their unique road safety issues. With this understanding, the study followed the tenets of community-based participatory research with an understanding that further action in addressing the unique safety issues would require consolidated efforts from various stakeholders including the communities of both parents and teachers [19]. Chirimba Primary is located within a densely populated peri-urban location of Chirimba, surrounded by unstructured housing and a busy, congested market. The school has approximately 5253 students, most of whom are from low-income households. Due to limited teaching facilities and teachers, students rotate between two school shifts: one group is taught in the morning and another in the afternoon. The other school—Kanjedza Primary—is located next to busy main roads in the structured urban area of Kanjedza. The school has a student population of approximately 564. Learners from this school predominantly come from middle-to-higher-income households as determined by Malawi’s standards of living.

### 2.3. Sampling and Data Collection

The sampling and data collection processes were guided by Onwuegbuzie et al.’s (2009) Qualitative Framework for Collecting and Analysing Data in Focus Group Research [20]. Participants in the FGDs were recruited with the support of the schools’ Head Teachers, who had previously collaborated with the researchers on other REmWalkS project interventions. Each Head Teacher was asked to identify a purposive sample of six teachers (three males and three females) and six parents (three males and three females) of children aged 12–16 years who were willing and available to participate in the study. This resulted in a total of 24 participants, with 12 teachers and 12 parents from both schools.

To ensure the triangulation of findings within the broader project, parents and teachers were selected based on their involvement in other aspects of the research such as the photovoice and the knowledge, attitude, and practices survey. Participants were also purposively chosen from different areas surrounding the schools to capture diverse lived experiences and perspectives. The researchers provided the Head Teachers with Participant Information Sheets and a written brief of the project to facilitate the recruitment process. The Head Teachers then provided a list of potential participants, which the researchers verified to ensure they met the inclusion criteria.

The rationale for this sample size was to include enough participants to yield diverse information while maintaining an environment conducive to open and comfortable sharing of perspectives [20]. An equal representation of females and males was maintained across all participant groups.

An FGD guide was designed by the researchers to ensure consistency across the discussions. The guide, developed after a systematic review of literature and expert input, was divided into three sections: travel to school, perceived risk of road traffic injuries, and exposure to air pollution. Each section began with general questions and progressively focused on specific issues. Discussions aimed to gain insights into participants’ perceptions as adults, guardians, and community members.

Data collection was conducted in August 2022 by trained research assistants and local researchers. Each FGD had one note-taker and one facilitator. Two FGDs were conducted in each school—one with all teachers and another with all parents—ensuring gender balance across groups. This setup allowed for a comprehensive understanding of the issues explored [21].

FGDs were held in isolated, secure classrooms to minimize distractions. Discussions were facilitated in Chichewa, Malawi’s local language, to promote inclusive and dynamic interactions. All FGDs were audiotaped with informed consent from participants, and each participant was assigned a number to protect their identity.

### 2.4. Data Management and Analysis

FGDs were transcribed and translated verbatim into English from Chichewa, whereafter they were reviewed and cleaned by two members of the research team (B.C., M.N.). Cleaned transcripts were then exported into NVivo data analysis software (version 12) for management of data, coding, and analysis. Transcripts were upload to NVivo and the coding was performed manually through the transcripts. The team employed a combination of deductive and inductive approaches to data analysis [22], with the domains and themes being constructed from the study objectives and the codes emerging from the data. A step-by-step process of thematic analysis to develop a conceptual model in qualitative research was used as approach for the thematic analysis [23]. Thematic content analysis was then used to synthesize the data, identifying relations, patterns, and explanations in the data. Two of the authors (B.C., M.N.) from Malawi, who were involved in conducting the FGDs and fluent in Chichewa, independently coded the data and compared their findings before the third author (A.P.) came in to arbitrate on the final codes and themes, facilitating consensus in the process. The independent engagement of all three ensured the enhancement of rigor and minimized bias during the process. The third author was in the United Kingdom and was not involved in conducting the FGDs, which provided an alternative perspective to the data analysis.

Audio files and transcriptions were uploaded to Teams-cloud shared folders only accessible to the research team. Upon completion of transcription, they were immediately deleted from recording devices.

## 3. Results

**Figure 1:**
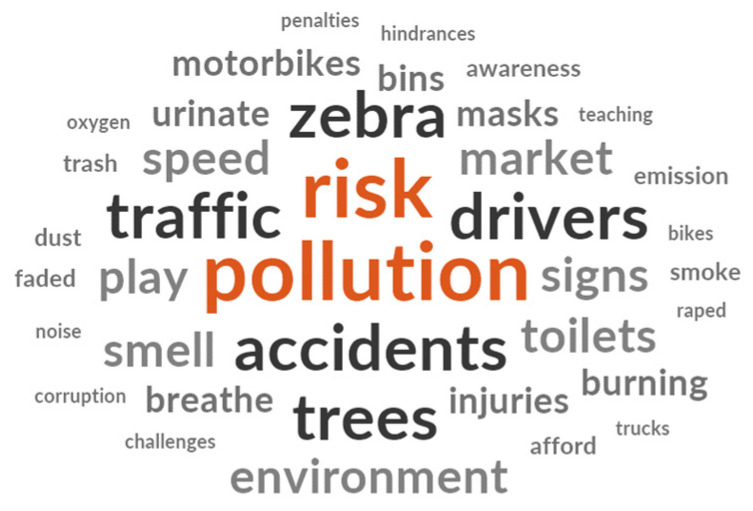
Word cloud from focus group discussions.

The FGDs explored the perceptions of parents and teachers regarding the risks that children face on their way to school. A visual word cloud representing the most commonly occurring words/themes that emerged from the transcripts was created using NVivo 12 (Figure 1). It was evident that words such as “pollution”, “risk”, “accidents”, “zebra”, “drivers”, “traffic”, and “trees” emerged prominently in the data analysis, signifying their frequent mentions during the discussions. Although some unrelated words appeared in the word cloud, further analysis of the surrounding text revealed that these words were connected to the community’s perceptions and concerns relevant to our research objectives. Notably, the word cloud also indicated potential misunderstandings about air pollution among participants.

Findings were categorized into two main domains: Road Safety and Air Pollution. The domains, themes, and codes were agreed upon by the research team and are shown in Table 1 below. It is important to mention that the themes were used throughout the project in other stages.

### 3.1. Road Safety Risk Factors

#### 3.1.1. Behavior of Children

The behavior of children was the most cited road safety risk factor. Parents and teachers observed risky behaviors among children, including being easily distracted, running or walking on the wrong side of the road, and playing on the streets—particularly football. These behaviors were perceived to contribute to children becoming distracted while navigating the road system. Additionally, activities like eating, reading, and listening to music with headphones were also mentioned. One teacher explained, “*Children play football while walking home on the road. This is very risky to them because if a ball lands on the road where cars are also passing, it can very easily be stepped on, and it can also cause unexpected accidents*” (Participant 7).

#### 3.1.2. Behavior of Parents

Teachers in Chirimba expressed concern about parental behavior, emphasizing the lack of supervision as children walk to school, particularly among the younger students. The agreed position was that the younger children need to be accompanied to school. However, the challenge was that “*many parents especially mothers are doing business there at the market and maybe do not find time to come with children to school, so children come on their own*” (Participant 7). Participants attributed the lack of parental supervision to the parents’ need to go to work, which often coincides with the start time of school.

Further, there were instances identified where parents encouraged risky behavior among groups of children walking from school. For example, one parent observed that “*Many children fight, especially when going to school, and many parents seem to encourage this practice*” (Participant 1).

#### 3.1.3. Behavior of Drivers

The poor behavior of drivers was another common risk factor mentioned in the discussions. Some of the concerns identified by both parents and teachers were the drivers’ use of phones while driving, as well as speeding, the consumption of alcohol, and the lack of respect for traffic signs such as the stop sign or the zebra crossing sign. One parent from Kanjedza remarked that “*Drivers do not do like they used to on the zebra cross, they can see that the child has entered the road, but they do not stop the car*” (Participant 18).

Speeding was identified as a major risk factor by both parents and teachers, especially during the morning peak hours. During this time, participants reported many drivers neglecting road signs and driving regulations, with one even suggesting it was “*…a competition of minibusses to get more customers*” or that *“sometimes it is the passengers who force drivers to speed because they say they are rushing to where they are going*” (Participant 7).

#### 3.1.4. Behavior of Motorcycle Riders

This was a concern commonly cited in the FGDs in Chirimba, where drinking alcohol and the lack of a license were the contributing factors to the risky behavior of motorcycle drivers. It was noted that “*a lot of motorcycle drivers do not have the training, so this is making them not know and follow rules regulating reckless driving*” (Participant 12).

Like with motor vehicle drivers, the participants were concerned with the speeds at which motorcyclists operate with little to no regard for pedestrians or their own passengers’ safety. This was raised in the context that “*many parents use motorbikes to drop their children off at school because they are looking for cheap transport*” (Participant 3), yet “*most of the motorbike operators have no licenses, no helmet and they are not familiar with the road safety laws*” (Participant 4).

#### 3.1.5. Infrastructure

There was a consensus among parents and teachers in Chirimba that the lack of adequate infrastructure to protect children walking to school and poor road maintenance were major risk factors. One teacher said “*The school is close to a market and often the road signs are removed. It would be important to replace road signs in time after they have been removed to make drivers aware next to them is a school*” (Participant 2).

The absence of speed bumps, clear zebra crossings, and road signs, in general, formed the basis of a participant’s perception that children are “*at a high risk on the road because our road network system is not that good*” (Participant 11).

Concerns regarding the lack of law enforcement emerged in the focus group discussion in Chirimba, where a participant emphasized, “*They are not safe because the police are supposed to protect the children. Instead, they block the cars to receive money*” (Participant 4).

#### 3.1.6. Personal Safety

The personal safety of children on their journeys to school was a topic that emerged frequently during both teachers’ and parents’ FGDs. Issues such as encountering street children or sexual assaults were discussed and played an important part in the discussions. While not strictly related to road safety as a concept or the dangers encountered due to traffic, the participants mentioned that to avoid these dangerous situations on their way to school, children would opt to walk on busier roads that potentially lacked adequate infrastructure such as in the form of sidewalks or crossings.

In Kanjedza, teachers were concerned about the safety of their children when they walked back from school because they went home alone. One teacher said, “*Coming to school, they are safe because teachers escort them, but when they are going home, they are not safe*” (Participant 20).

### 3.2. Road Safety Interventions

#### 3.2.1. Infrastructure

Participants in both FGDs had suggestions on how the infrastructure could be improved, and both agreed on the need to place road humps in front of the schools to decrease the speeds of vehicles.

In Chirimba, parents agreed that a zebra crossing was needed at the school, and teachers agreed, stating “To put signposts on our roads because through these children will know what to do when crossing the road and this too will make easy for drivers to be able to know what is ahead of them to avoid accidents on the road” (Participant 11).

There was already a zebra crossing in Kanjedza; however, it had faded, and participants suggested that it be repainted.

#### 3.2.2. Students’ Education

Students’ education was an intervention cited in all FGDs. Both parents and teachers suggested teaching road safety in school, encouraging children to travel in groups with older children, and introducing school patrols.

In Chirimba, one of the initiatives proposed included urging parents to follow up on their children to ensure that they arrived safely at school.

Kanjedza’s discussions focused on ideas surrounding the most useful tools to teach road safety. For example, one teacher suggested that “*there is a song they sing to remind them of how best to walk near roads*” (Participant 20). Teachers identified the need to educate all road users, including drivers and motorcyclists, not just schoolchildren.

#### 3.2.3. Legislation, Policy, and Enforcement

This topic was discussed in Chirimba, with parents and teachers agreeing that the government should establish strict(er) laws for the use of motorcycles, that people without training should not be allowed to ride, and that there should be strict(er) penalties for people who do not respect road signs.

Parents and teachers suggested the need for stricter laws around schools in Chirimba. In both schools, participants suggested the need for humps to reduce the speeds around the schools.

### 3.3. Air Pollution Risk Factors

The risk factors regarding air pollution were classified into two categories: environmental risks and air pollution. It became clear that participants commonly misperceived unpleasant odors as synonymous with air pollution. Many of the participants believed that air pollution risks were associated with “bad smells”, and for that reason, these were categorized as environmental risks.

#### 3.3.1. Environmental Risks

A common discussion point regarding environmental risks was the concern about bad practices by people and the inappropriate management of garbage by public services. For example, one teacher suggested that “*During the night people wake up and take their trash and throw it anywhere but not in bins for proper disposal and also a lot of trash is being thrown in streams which is also contaminating the water and at the same time producing a bad smell to the people around so there is a need to put proper disposable bins*” (Participant 8).

Other concerns were the burning of waste, the disposal of waste into the river, the misuse of toilet facilities, and concerns regarding the spread of diseases by polluted water. “*Imagine children walking barefoot on such garbage water. It is unhealthy and leads to diseases like cholera and bilharzia*”, remarked a teacher (Participant 24).

#### 3.3.2. Air Pollution

Participants mentioned pollution from cars and dusty roads, citing health concerns. For example, one parent suggested that “*Many children are breathing bad air that can cause diseases such as mold diseases, coughing, lung diseases, and tuberculosis*” (Participant 6).

### 3.4. Air Pollution Interventions

#### 3.4.1. Enforcement

Teachers in Chirimba thought that rules regarding garbage disposal should be enforced. Participants emphasized the need to involve local chiefs in eliciting action among members of the community, as one parent suggested: “*Chiefs can also be reached on this matter since they are their people, and they can see what they can do*” (Participant 17).

#### 3.4.2. Infrastructure

An intervention cited in all FGDs was the planting of trees around the schools. In Kanjedza, a teacher suggested that “*Relocating industries to areas less populated*” (Participant 20) would help reduce air pollution.

#### 3.4.3. Legislation

Participants proposed various legislative options including restricting tree cutting.

Land regulations emerged in Chirimba, where one participant suggested, “I see that the authorities, for example, Chiefs, when selling land to people, they do not leave a distance in between the plots and also our leaders have the duty to control how people litter trash as people who have power in the community. So, I think when Chiefs sell land, they should make sure there is a distance e.g., two meters from one plot to another to give space for a path and toilets to be away from each other so that bad smells should not be too much as it is now…they must also teach people to plant trees because good air comes from trees” (Participant 9).

#### 3.4.4. Public Awareness/Education

Participants suggested that children could be encouraged to wear masks while walking to school, that the community be taught how to correctly dispose of garbage and observe good hygiene practices, and that toilet owners empty them when became full. One teacher remarked that there was “*a need for the students, through education, to know that bad smell or polluted air is not good, and they have the responsibility to protect their school from air pollution. For example, if they see anyone urinating on the walls, they must report that to the authorities who can take action on the person doing that practice*” (Participant 9).

## 4. Discussion

This study was part of the main REmWalkS research project, which aimed to develop a package of road safety and air pollution interventions to improve child safety. It used focus group discussions to engage with parents and teachers to better understand children’s risks on their walks to school in Blantyre, Malawi. It also sought to understand the community’s perspectives on possible interventions to reduce both the risk of road traffic collisions and exposure to poor air quality.

In their characterization of a road safety risk, participants emphasized risky behaviors by different actors, including the children themselves, motor vehicle drivers, and motorcycle operators, as common road safety risk factors. They further identified the role of parental neglect and the authorities’ failure to improve and maintain road infrastructure in amplifying the risks.

The children’s risk of exposure to air pollution was often associated with “bad smells” resulting from poor practices such as burning waste, the disposal of waste into rivers and streams, the poor management of pit latrines in the homes, and the inappropriate management of garbage by public service providers. Participants perceived interventions to reduce risks that revolved around both education and the sensitization of different actors, legislation and policy development, the enforcement of penalties through government authorities and local chiefs, and enhancing infrastructure. To the best of our knowledge, this was one of the first studies to qualitatively assess community perceptions surrounding both road safety and air pollution. Addressing these factors is crucial as both significantly impact the health, safety, and overall quality of life for a community. Ensuring safer roads and cleaner air is essential, especially for children commuting to schools, as it directly influences their physical well-being, cognitive development, and ability to learn and thrive in a healthy environment.

**Figure 2:**
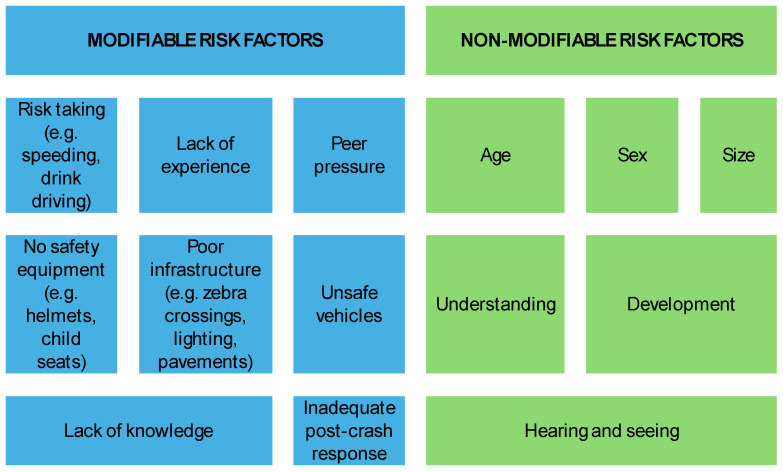
Road traffic injuries: risk factors among children and adolescents.

Road safety risks in children and adolescents can be categorized into non-modifiable and modifiable risk factors (Figure 2) [24]. The risk factors identified by participants in this study fell under the modifiable category. These included behaviors influenced by a lack of knowledge and experience, as well as by peer pressure, which could lead to risky actions. Additionally, poor infrastructure was specifically highlighted as a risk factor in this study.

Participants in the study described the playful behavior of children on their walks to school, raising concerns over their ability to consider their safety as they walked on the roads. This risk can be categorized into that of risks taken [24]. For the younger children, this was associated with a lack of parental supervision on the walk to school. This was similar to a study from Pakistan, which characterized the risk of safety by noting that children walking without the company of adults are less aware of the danger, and this, together with the fact that children’s narrower peripheral vision is about one-third of that of adults, makes it difficult for them to estimate vehicle speed accurately [25].

Driver behavior was one of the most commonly cited causes of road traffic incidents in low-income countries due to dangerous practices on the roads [26]. Participants in this study were particularly concerned about public transport operators, including minibus drivers and motorcycle taxi operators, citing their lack of respect for road signs, speeding, and alcohol consumption while driving as major risk factors. Participants further shared their experiences on the effects of motor vehicle drivers’ use of mobile phones while driving, raising concerns about the drivers’ reduced concentration and awareness of road signs and signals and children’s actions. The use of mobile phones while driving has been closely linked to motor vehicle accidents [27] and could be responsible for 25–50% of collisions, with the potential to triple the risk of road crashes [28].

The participants highlighted the behavior of authorities regarding some questionable practices within the city as a significant concern. This issue has been identified as a barrier to achieving road safety goals as evidenced by findings from a modified Delphi study conducted in Ghana [29]. Ethical issues from authorities have also emerged as a prominent theme in studies conducted in Ghana and Kenya, even without direct questioning on the topic [29,30], similar to what was found in our study, where ethical issues from authorities were discussed during the sessions. Authorities’ questionable practices play a crucial role in road safety, reducing the effectiveness of road safety regulations and their enforcement [30].

The concerns expressed by parents and teachers regarding infrastructure deficiencies, such as the lack of zebra crossings, signage, and road humps, demonstrated that parents’ perceptions of road safety are closely tied to the infrastructure surrounding schools, including features such as sidewalks, road crossings, the presence of crossing patrols or guards, vehicle speeds, traffic volumes, tree coverage along routes, and neighborhood amenities [31,32,33]. These shared concerns underscore the importance of addressing specific infrastructure issues to enhance overall road safety perceptions in the community.

Parents and teachers highlighted personal safety as a significant concern, expressing fears about potential risks such as assault during the journey to school. Research supports these concerns, showing that issues related to general neighborhood safety, encounters with dangerous animals, violence and crime, and interactions with unfamiliar adults along the walking route are among the worries parents have regarding their children’s safety [32,34]. Additionally, the built environment and crime-related safety have been identified as barriers to active commuting to school [35,36].

These concerns cited by teachers and parents are closely tied to the most common intervention suggested in this study—students’ education. This intervention is directly linked to addressing the primary concern surrounding road safety risks, particularly focusing on the behavior of individuals involved in road safety. However, a more holistic approach is necessary; in addition to education, we must consider infrastructure improvements, policy enforcement, and community engagement. Educational initiatives should be tailored to the specific needs of different groups considering variations in gender and age [37]. Research emphasizes the critical role of comprehensive road safety strategies, which include education, improved road infrastructure, stricter law enforcement, and community participation, given their proven association with reducing risky behaviors and promoting responsible road use [38,39,40,41]. Taking into account the fact that most children do not look left and right before crossing a road, children might forget to check for traffic before crossing the road, assuming no cars are coming, even when there could be [25,33]. For this reason, education-focused initiatives should prioritize enhancing the understanding of traffic rules and signals, road risk perception, and self-reported road behaviors [38].

Education programs conducted for children and parents have been reported to be successful in different studies, with some of them looking to change parents’ behavior through children’s education [37,42]. However, it is important to highlight that children acquire knowledge through observations, and their behaviors, habits, and safe patterns are learned from their figures of authority [43].

The evidence reveals a prevalent emphasis on interventions to change user behavior through enforcing penalties, traffic laws, and educational programs [44]. However, in low- and middle-income countries (LMICs), interventions tend to adopt single-component approaches with a narrow focus, often overlooking critical contextual factors contributing to road safety challenges. Collisions are frequently attributed solely to road users as the primary changeable elements [44,45]. By incorporating community input and understanding local contexts, interventions can be tailored to address specific challenges and achieve more impactful and sustainable improvements in road safety outcomes.

Throughout the FGDs, participants demonstrated an awareness of existing interventions to enhance road safety. The conversations predominantly centered around offering valuable suggestions for improving and optimizing existing road safety measures and strategies already implemented within the community. This focus highlights the participants’ recognition of the effectiveness of existing interventions and their belief that refining these measures is the most practical approach to addressing road safety challenges.

When considering air pollution risks, it became evident that participants had a limited understanding of the actual sources contributing to air pollution. This is supported by published literature describing how individuals associate bad smells and dirtiness with air pollution [46,47]. While people are generally aware of the consequences of air pollution, there is often a lack of clear knowledge about what air pollution entails [46].

The risk factors identified in this study are consistent with findings from other studies. Participants expressed a primary concern about odors emanating from public bathrooms and trash, which also was identified in another study conducted with women in Nepal, who reported “bad smells” as sources of pollution [47]. Interestingly, the FGDs did not discuss car pollution; however, individuals are deeply concerned about the health impacts of air pollution, especially respiratory diseases.

Furthermore, other research indicates that individuals with higher levels of education tend to report higher levels of annoyance or demonstrate a greater awareness of environmental issues like air pollution. This highlights the influence of education on the perceptions and understanding of air quality concerns [46,48].

The proposed interventions for addressing air pollution in the FGDs were tailored to the identified risk factors. Like road safety discussions, education emerged as the most cited intervention; however, this education focused on promoting the use of masks, proper garbage disposal practices, and good hygiene habits.

Based on these findings, it is evident from the community voice that interventions at this stage should prioritize optimizing children’s walking routes to and from school, aiming to minimize exposure to congested roads and ensure safe passage. Studies have shown that children attending schools located near highways face increased risks of respiratory diseases and neurological disorders [12]. One proposed intervention involved relocating industries situated close to schools to mitigate exposure.

The way children commute to school significantly impacts their exposure to air pollution, with traffic congestion playing a pivotal role. Factors such as waiting times at road crossings and walking alongside busy roads contribute to increased exposure [6,7]. Despite these critical considerations, there remains a lack of literature on this topic within low-income countries, underscoring the urgent need to enhance research efforts in this area.

## 5. Conclusions

This paper highlights the urgent need for comprehensive strategies addressing road safety and air pollution in the context of children’s commutes to school. Moving forward, future research should focus on bridging knowledge gaps and evaluating the effectiveness of interventions aimed at improving road safety and reducing air pollution exposure during school travel. It underscores the importance of adopting multidisciplinary approaches encompassing education, infrastructure development, policy enforcement, and community engagement. Policymakers should prioritize initiatives that enhance road safety measures, promote sustainable transportation options, and mitigate sources of air pollution along school routes. Additionally, addressing this issue across different sectors is crucial. Urban planning must be integrated into the road safety and climate change agenda. Ensuring personal security and reducing violence among children are essential to guaranteeing safe walks to school. It is also important to consider the socio-economic context, which allows parents, especially mothers, to have better opportunities to devote time to their children’s school commutes.

Future research should explore innovative solutions and technologies to reduce traffic congestion, promote active transportation, and enhance air quality monitoring. Additionally, policies should prioritize investments in safe and accessible infrastructure such as pedestrian-friendly pathways and green transportation options.

Ultimately, by integrating these research findings into policy and practice, we can create safer and healthier environments for children traveling to school, ensure their well-being, and foster sustainable communities.

## Figures and Tables

**Table 1:** Domains, themes, and codes.

Domains	Themes	Codes
Road Safety	Risk Factors	Behavior of children
		Behavior of drivers
		Behavior of motorcycle riders
		Behavior of parents
		Infrastructure
		Personal safety
	Interventions	Infrastructure
		Student’s education
		Legislation/policy and enforcement
Air Pollution	Risk factors	Environmental risks
		Air pollution
	Interventions	Enforcement
		Infrastructure changes
		Legislation
		Public awareness/education

## Data Availability

The data presented in this study will be available on request from the corresponding author once all related papers have been published. The data are not publicly available due to privacy restrictions.
